# Characterization of Rv0888, a Novel Extracellular Nuclease from *Mycobacterium tuberculosis*

**DOI:** 10.1038/srep19033

**Published:** 2016-01-08

**Authors:** Guanghui Dang, Jun Cao, Yingying Cui, Ningning Song, Liping Chen, Hai Pang, Siguo Liu

**Affiliations:** 1 Division of Bacterial Diseases, State Key Laboratory of Veterinary Biotechnology, Harbin Veterinary Research Institute, Chinese Academy of Agricultural Sciences, 427 Maduan Street, Nangang Dist, Harbin 15000, PR China; 2 School of Medicine, Tsinghua University, Beijing, 100084, China

## Abstract

Bacterial extracellular nucleases play important roles in virulence, biofilm formation, utilization of extracellular DNA as a nutrient, and degradation of neutrophil DNA extracellular traps. However, there is no current data available for extracellular nucleases derived from *M. tuberculosis*. Herein, we have identified and characterized Rv0888, an extracellular nuclease in *M. tuberculosis.* The protein was overexpressed in *E. coli*, and the purified Rv0888 protein was found to require divalent cations for activity, with an optimal temperature and pH of 41 °C and 6.5, respectively. Further results demonstrated that Rv0888 nuclease activity could be inhibited by four Chinese medicine monomers. Based on sequence analysis, Rv0888 nuclease exhibited no homology with any known extracellular nucleases, indicating that Rv0888 is a novel nuclease. Site-directed mutagenesis studies revealed that the H353, D387, and D438 residues play catalytic roles in Rv0888. *In vivo* infection studies confirmed that Rv0888 is required for infection and is related to pathogenicity, as the persistent ability of recombinant *Mycobacterium smegmatis* (rMS) Rv0888NS/MS and Rv0888S/MS is significantly higher than pMV262/MS in the lung tissue, and the Rv0888NS/MS and Rv0888S/MS could produce pathological changes in the mice lung. These results show that Rv0888 is relevant to pathogenicity of *M. tuberculosis*.

A variety of bacteria produce extracellular DNases/nucleases that are important for virulence, biofilm formation, utilization of extracellular DNA as a nutrient, and degradation of neutrophil DNA extracellular traps (NETs). Extracellular nucleases produced by *Shewanella*, *Serratia marcescens*, *Staphylococcus aureus* allow these bacteria to degrade extracellular DNA to its basic components: phosphate, carbon, and nitrogen^1^,^2^,^3^. A recent study by Seper *et al.* demonstrated that wild-type *Vibrio cholerae* rapidly degraded the DNA component of NETs through the combined activity of two extracellular nucleases, Dns and Xds^4^. In *S. aureus*, an extracellular nuclease also degraded NETs, triggering caspase-3-mediated death of immune cells^5^. Moreover, nuclease A (Gbs0661), an extracellular nuclease of *Streptococcus agalactiae*, is required for full virulence of the organism^6^ and attacks NETs, which are a component of the host defense response that immobilizes microbes for subsequent clearance by innate immunity machinery, including phagocytosis by macrophages. A Mn^2+^ -stimulated deoxyribonuclease (Nuc B) is released by *Bacillus subtilis* 168 during sporulation in glucose-deficient medium, which degrades structurally important nucleic acids, thereby controlling the development and dispersal of bacterial biofilms^7^,^8^.

*Mycobacterium tuberculosis* (*M. tuberculosis*) is a Gram-positive bacterium that causes tuberculosis (TB), which remains one of the world’s deadliest communicable diseases. In 2013, an estimated 9.0 million people contracted TB and a subsequent 1.5 million people died from the disease, of whom 360,000 were HIV-positive^9^. *M. tuberculosis* is phagocytosed by alveolar macrophages and dendritic cells after inhalation into the lung. However, *M. tuberculosis* can proliferate within these immune cells, eventually escaping from the phagosome and migrating to draining lymph nodes to spread the infection^10^,^11^,^12^. Rv0888, a protein that belongs to the large endonuclease/exonuclease/phosphatase family (Pfam family PF03372)^13^, has sphingomyelinase activity that has been detected in *M. tuberculosis* culture filtrates^14^. In this study, we identified and characterized Rv0888, the first extracellular nuclease to be reported from *M. tuberculosis*. We demonstrate that this enzyme is highly active in degrading different types of nucleic acids (chromosomal DNA, double-stranded PCR products, plasmid DNA, and baker’s yeast RNA) utilizing divalent metal cations (Ca^2+^ and Mn^2+^). In addition, the nuclease activity of Rv0888 is inhibited by four Chinese medicine monomers (Oleuropein, 6-Gingerol, Corylifolinin, and Acteoside). Finally, we confirmed that Rv0888 is relevant to mycobacterial pathogenicity.

## Results

### Expression and Purification of Rv0888

**Figure 1:**
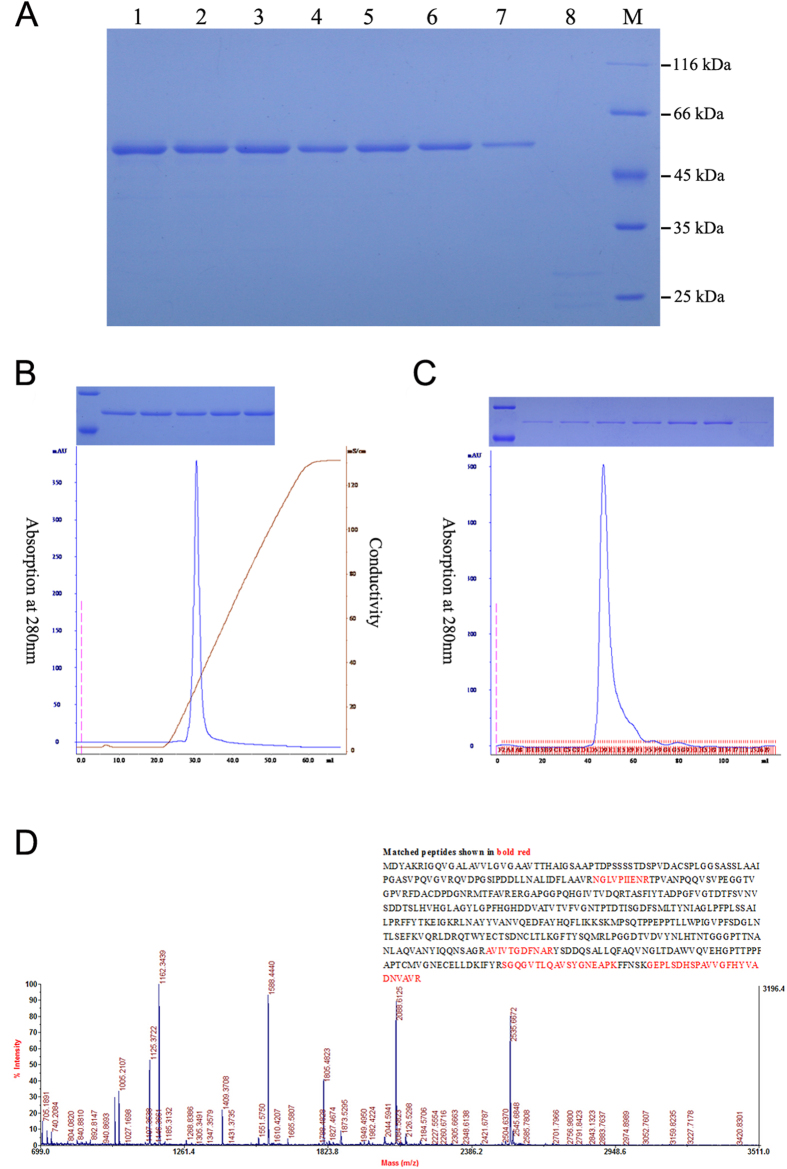
Recombinant Rv0888 purification and mass spectrometry identification. **(A)** SDS-PAGE analysis of affinity-purified Rv0888 protein. Purified Rv0888 protein was eluted using 20 mM Tris-HCl-150 mM NaCl-10% glycerol with an imidazole (pH 7.5) concentration gradient. Line 1: 500 mM imidazole; Line 2: 200 mM imidazole; Line 3: 100 mM imidazole; Line 4: 80 mM imidazole; Line 5: 70 mM imidazole; Line 6: 60 mM imidazole; Line 7: 40 mM imidazole; Line 8: 20 mM imidazole; Line M: protein molecular weight marker. **(B)** Rv0888 protein purified by ion exchange chromatography. **(C)** Rv0888 protein purified by gel filtration chromatography. **(D)** Matrix-assisted laser desorption/ionisation time-of-flight mass spectrometry peptide mass fingerprint spectrometry of the Rv0888 protein. Protein sequences that matched identified peptides are bolded and red.

The SignalP server revealed a signal sequence (1 to 31 residues) in the Rv0888 amino acid sequence and, as this hydrophobic sequence might increase the difficulty of expression, the fragment of *rv0888* from *M. tuberculosis* H37Rv was cloned without the predicted signal sequence. In order to purify the protein, Rv0888 was expressed as a 6 × His-tagged protein in *E. coli*. Though it was expressed in inclusion body form, Rv0888 was renatured and purified via a Ni^2+^ affinity chromatography resin (Fig. 1A). Then, the eluted Rv0888 protein was loaded onto an anion exchange chromatography column (Fig. 1B) and subsequently passed through a gel filtration chromatography resin, subsequently resulting in greater than 98% protein purity (Fig. 1C).

### Rv0888 protein identity confirmation

The purified Rv0888 protein was analyzed by mass spectrometry and peptide mass fingerprinting results were submitted to Mascot and used to search the SwissProt database. The analysis returned 10 results, with a score of 78 assigned to the expected protein Rv0888 (GeneBank Accession No. NP_215403.1) from *M. tuberculosis* H37Rv (Fig. 1D).

### Rv0888 nuclease activity specificity

**Figure 2:**
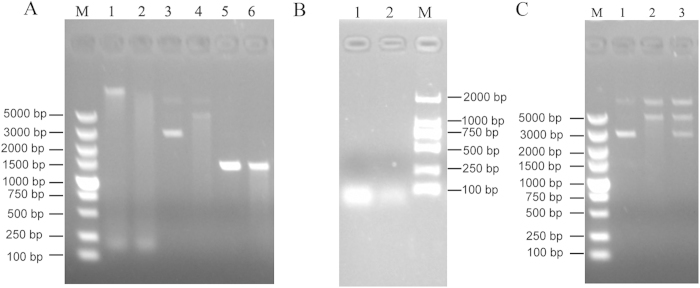
Digestion of various nucleic acids by purified Rv0888. The reaction was performed in 20 mM Tris-HCl pH 7.5 and 5 mM MgCl_2_ for 1 h at 37 °C. (**A**) Digestion of various DNA with purified Rv0888. Line M: DL5000 DNA Marker; Line 1: chromosomal DNA in 20 mM Tris-HCl (pH 7.5); Line 2: chromosomal DNA and purified Rv0888; Line 3: circular plasmid DNA in 20 mM Tris-HCl (pH 7.5); Line 4: circular plasmid DNA and purified Rv0888; Line 5: linear dsDNA in 20 mM Tris-HCl (pH 7.5); Line 6: linear dsDNA and purified Rv0888. (**B**) Digestion of RNA with purified Rv0888. Line M: DL5000 DNA Marker; Line 1: baker’s yeast RNA in 20 mM Tris-HCl (pH 7.5); Line 2: baker’s yeast RNA and purified Rv0888. **(C)** DNase activity requires cations. Line M: DL5000 DNA Marker; Line 1: circular plasmid DNA in 20 mM Tris-HCl (pH 7.5); Line 2: circular plasmid DNA and purified Rv0888 with 5 mM CaCl_2_ and 5 mM MnCl_2_; Line 3: circular plasmid DNA and purified Rv0888 with 5 mM CaCl_2_, 5 mM MnCl_2_ and 20 mM EDTA.

To verify the nuclease activity, purified Rv0888 was incubated with different nucleic acids, including linear dsDNA (PCR production), circular plasmid DNA (pGEX-6p-1 vector), chromosomal DNA (*E. coli* DNA) or RNA from baker’s yeast. Surprisingly, all of the nucleic acids were degraded by the Rv0888 protein (Fig. 2A,B). These results indicated that Rv0888 is a non-specific nuclease.

### Effect of divalent cations and metal chelators on Rv0888 activity

The effect of different divalent cations on nuclease activity of Rv0888 was evaluated. In the absence of divalent cations, nuclease activity was not detected. The enzymatic activity was optimal in the presence of 5 mM CaCl_2_ and 5 mM MnCl_2_. Other divalent cations salts–CaCl_2_, MgCl_2_, BaCl_2_ and NiCl_2_–were demonstrated to display different stimulation effects of Rv0888 activity, and Rv0888 activity was fully inhibited by 20 mM EDTA (Table 1; Fig. 2C).

### Effect of pH and temperature on Rv0888 activity

**Figure 3:**
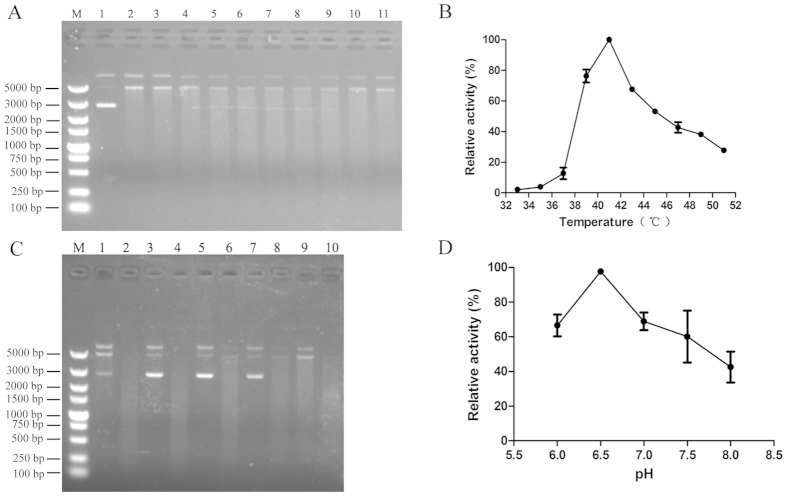
Effect of pH or temperature on Rv0888 enzymatic activity. (**A**) Analysis of the effect of temperature on Rv0888 enzymatic activity by agarose gel electrophoresis. The reaction was performed in 20 mM Tris-HCl pH 6.5, 5 mM CaCl_2_ and 5 mM MnCl_2_ for 1 h. Line M: DL5000 DNA Marker; Line 1: circular plasmid DNA; Lines 2-11: circular plasmid DNA and Rv0888 ranging between 33 °C and 51 °C at 2 °C intervals. (**B**) Quantification of DNase activity by spectrophotometry and error bars are given as standard deviations. (**C**) Analysis of the effect of the pH on Rv0888 enzymatic activity by agarose gel electrophoresis. The reaction was performed in 20 mM Tris-HCl, 5 mM CaCl_2_ and 5 mM MnCl_2_ for 1 h at 37 °C. Line M: DL5000 DNA Marker; Lines 1, 3, 5, 7, 9: circular plasmid DNA ranging between pH 6.0 and pH 8.0 at 0.5 intervals; Lines 2, 4, 6, 8, 10: circular plasmid DNA and Rv0888 ranging between pH 6.0 and pH 8.0 at 0.5 intervals. (**D**) DNase activity was quantified by spectrophotometry and error bars are given as standard deviations.

The effects of pH and temperature on Rv0888 activity towards circular plasmid DNA are displayed in Fig. 3A,C, respectively. Rv0888 had full activity across a pH range of 6.0-8.0, with maximal activity at pH 6.5 (Fig. 3D). Measurements of Rv0888 activity across a wide temperature range indicated that the optimal temperature for the Rv0888 was 41 °C (Fig. 3B).

### Kinetic studies of Rv0888 activity

**Figure 4:**
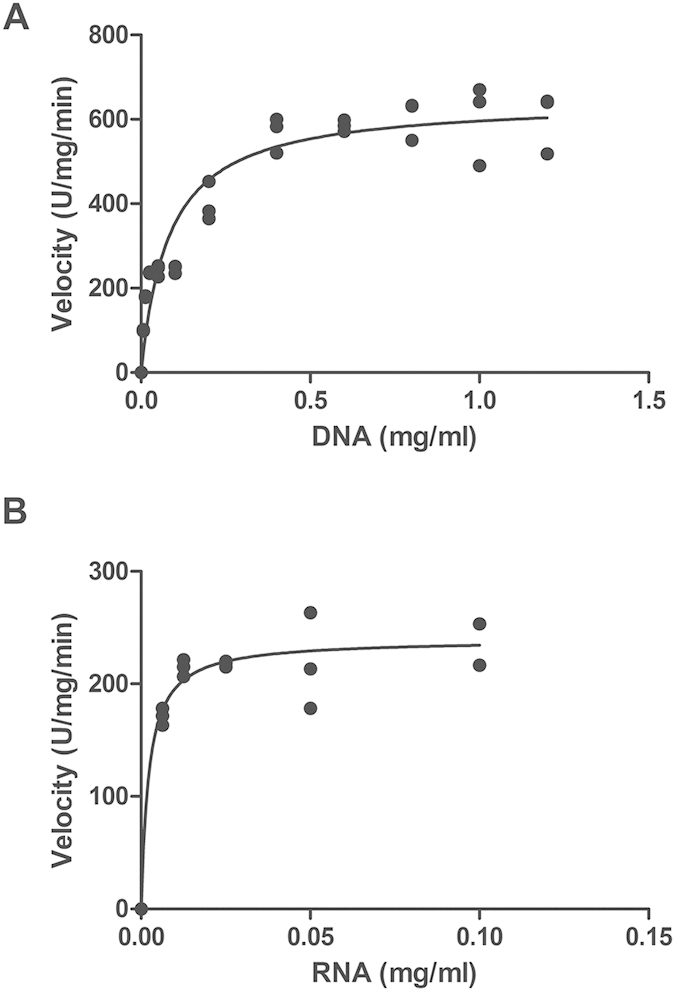
Michaelis-Menton kinetics assays of the DNase (**A**) and the RNase (**B**) activity of Rv0888. The reaction was performed in 20 mM Tris-HCl pH 6.5, 5 mM CaCl_2_ and 5 mM MnCl_2_ for 1 h at 41 °C. *K*_m_ values for DNA and RNA were 0.306 ± 0.04 mg/mL and 0.012 ± 0.01 mg/mL, respectively; the V_max_ values for DNase and RNase activity were 600.56 U/mg/min and 241.11 U/mg/min, respectively.

The kinetic parameters of the Rv0888 nuclease were determined at 41 °C, pH 6.5 and including 5 mM divalent cation salts (CaCl_2_ and MnCl_2_) with two different substrates: calf thymus DNA and baker’s yeast RNA. Michaelis-Menton kinetics assays revealed that the *K*_m_ values toward DNA and RNA were 0.306 ± 0.04 mg/mL and 0.012 ± 0.01 mg/mL, respectively (Fig. 4A,B). V_max_ values were found to be 600.56 U/mg/min and 241.11 U/mg/min for deoxyribonuclease and ribonuclease activities, respectively (Fig. 4A,B).

### Rv0888 inhibitors

**Figure 5:**
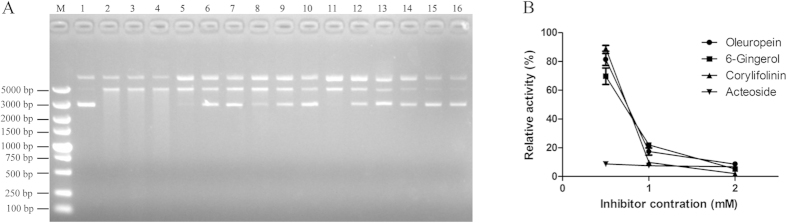
Oleuropein, 6-Gingerol, Corylifolinin, and Acteoside inhibit Rv0888 activity against circular plasmid DNA. The reaction was performed in 20 mM Tris-HCl pH 6.5, 5 mM CaCl_2_ and 5 mM MnCl_2_ for 1 h at 41 °C. **(A)** Line M: DL5000 DNA Marker; Line 1: circular plasmid DNA; Line 2: circular plasmid DNA and Rv0888; Line 3: circular plasmid DNA and Rv0888 in 50% ethanol; Line 4: circular plasmid DNA and Rv0888 in 50% dimethyl sulfoxide; Lines 5–7: circular plasmid DNA, Rv0888 and Oleuropein (0.5 mM, 1 mM, 2 mM, respectively); Lines 8–10: circular plasmid DNA, Rv0888 and 6-Gingerol (0.5 mM, 1 mM, 2 mM, respectively); Lines 11–13: circular plasmid DNA, Rv0888 and Corylifolinin (0.5 mM, 1 mM, 2 mM, respectively); Lines 14–16: circular plasmid DNA, Rv0888 and Acteoside (0.5 mM, 1 mM, 2 mM, respectively). **(B)** Quantification of DNase activity by spectrophotometry and error bars are given as standard deviations.

The nuclease activity of Rv0888 was inhibited by four Chinese medicine monomers (Oleuropein, 6-Gingerol, Corylifolinin, and Acteoside). As showed in Fig. 5A,B, the inhibition effect was concentration-dependent.

### Site-Directed Mutagenesis

**Figure 6:**
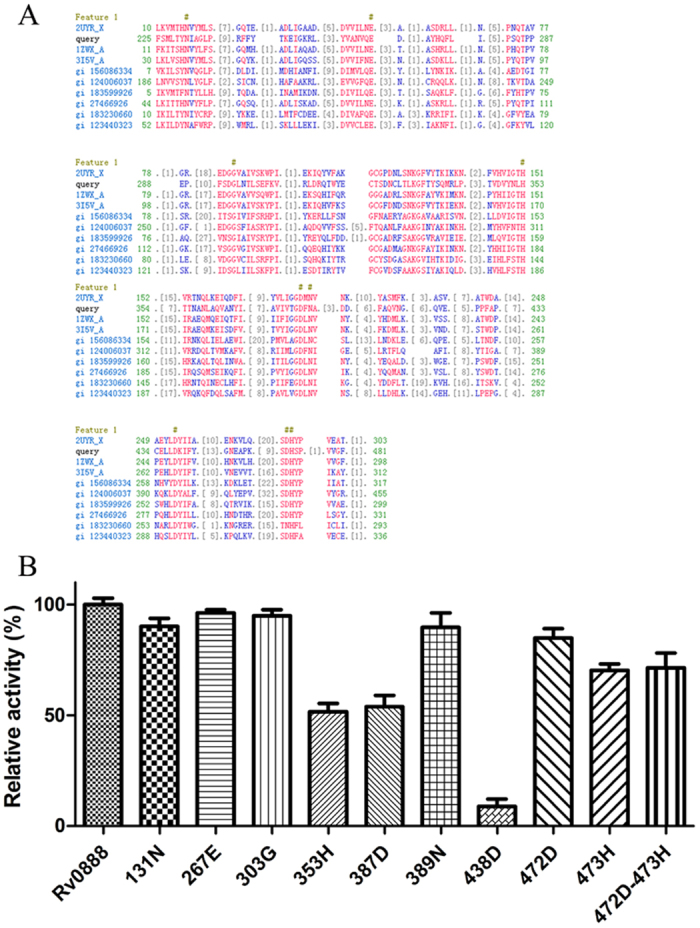
Site-directed mutagenesis of *Rv0888*. Ten amino acid residues were substituted in Rv0888 by performing site-directed mutagenesis on the gene: N131A, E267A, G303A, H353A, D387A, N389A, D438A, D472A, H473A, D472A-H473A. **(A)** Multiple sequence alignment of Rv0888 with other endonuclease/exonuclease/phosphatase family members. Query: Rv0888; #: conserved residue. **(B)** The experiment was performed in 20 mM Tris-HCl pH 6.5, 5 mM CaCl_2_ and 5 mM MnCl_2_ for 1 h at 41 °C. Error bars are given as standard deviations.

Multiple sequence alignment of Rv0888 with related homologs indicated that nine targeted residues (N131, E267, G303, H353, D387, N389, D438, D472, and H473) were highly conserved in the endonuclease/exonuclease/phosphatase family (Fig. 6A). To identify residues in Rv0888 that are necessary for catalytic activity, specific codons corresponding to the important residues were altered by site-directed mutagenesis.

Ten protein variants were produced, each with a single residue of the conserved residues (N131, E267, G303, H353, D387, N389, D438, D472, H473, and D472-H473) replaced with alanine. Alanine was chosen for substitution, as it lacks a bulky side chain and therefore would be unlikely to disrupt the main chain conformation. Comparison of the variant enzyme to wild-type Rv0888 demonstrated that the D438A substitution resulted in a 92% loss of activity, the H353A and D387A substitutions resulted in a ~50% loss of activity, and both the H473A and D472A-H473A substitutions resulted in a ~30% loss of activity. The N131A, E267A, G303A, N389A, and D472A substitutions resulted in losses in activities of 10%, 4%, 6%, 11%, and 15%, respectively (Fig. 6B).

### Role of Rv0888 in *M. smegmatis* persistence in lung and histopathological analysis

**Figure 7:**
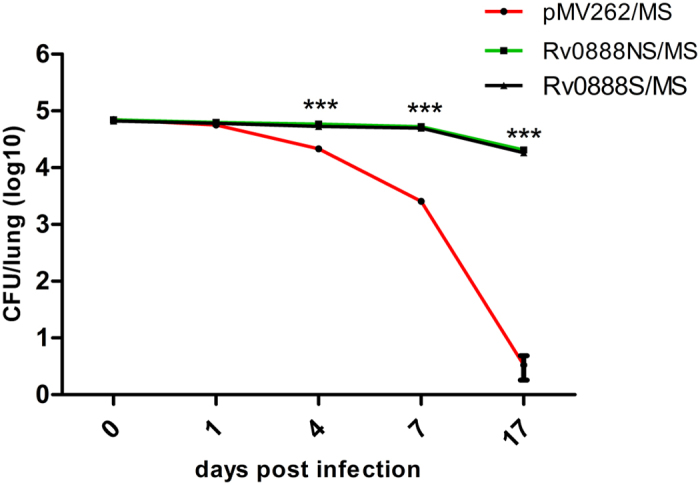
Presence of persistent recombinant *M. smegmatis* in mouse lung. Bacterial loads in infected lung tissue from BALB/c mice, as transmitted by intranasal infection with rMS pMV262/MS, Rv0888NS/MS and Rv0888S/MS were determined at 4 h, 24 h, 4 d, 7 d, and 17 d after infection.

The lungs are a portal to infection by *M. tuberculosis*, which can adhere to and invade lung epithelial cells^15^. In order to determine the role of Rv0888 in mycobacterial pathogenicity, the persistence ability of *M. smegmatis* overexpressing Rv0888 in lung tissue was estimated. Three groups of 3 mice were infected intranasally with a dose (2 × 10^7^ colony forming units) of the rMS strains pMV262/MS, Rv0888NS/MS, and Rv0888S/MS, respectively. Bacterial loads in lung tissue were measured at 4 h, 24 h, 4 d, 7 d, and 17 d after infection (Fig. 7). No significant difference was observed between the bacterial loads of Rv0888NS/MS and Rv0888S/MS groups at all time-points, whereas the bacterial loads of Rv0888NS/MS and Rv0888S/MS groups were remarkably higher, compared with that of pMV262/MS group, at 4 d, 7 d, and 17 d after infection. Importantly, in contrast to the almost total clearance of bacteria in the lungs of infected mice in the pMV262/MS group at 17 d, bacterial loads still persisted in mice of the Rv0888NS/MS and Rv0888S/MS groups.

**Figure 8:**
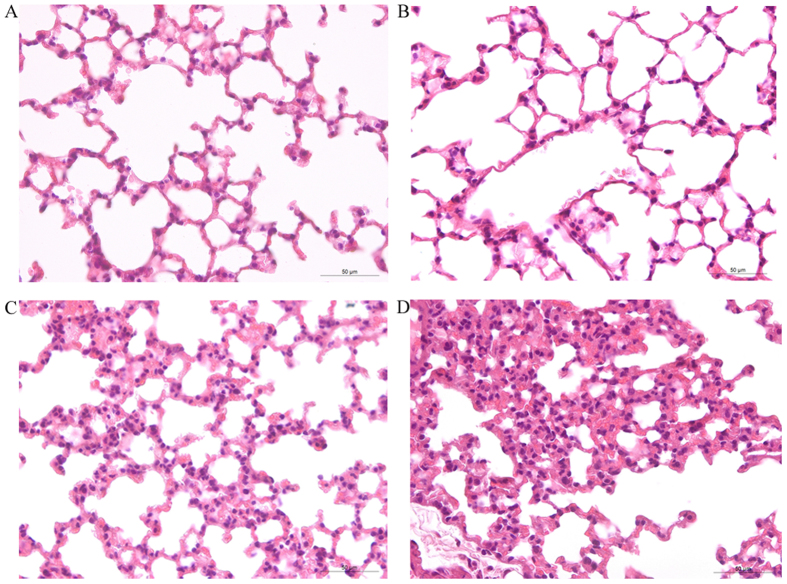
Histopathological analysis of mouse lung. BALB/c mice were infected by intranasal infection with rMS pMV262/MS, Rv0888NS/MS and Rv0888S/MS at 7 d, using PBS as a negative control. **(A)** PBS control; no pathological changes. **(B)** pMV262/MS; no pathological changes. **(C)** Rv0888NS/MS; mild hyperplasia of alveolar epithelial cells. **(D)** Rv0888S/MS; partial mild extravasated blood and hyperplasia of alveolar epithelial cells.

Histopathological analysis revealed that lungs from mice at 7 d after infection in the pMV262/MS group had no pathological changes, whereas mild hyperplasia was observed in alveolar epithelial cells of the mice in the Rv0888NS/MS group, and partial mild hematopedesis and hyperplasia were observed in alveolar epithelial cells in the Rv0888S/MS group (Fig. 8). Taken together, it was suggested that nuclease activity is required for mycobacteria to resist defensive clearance of lung tissue and may be related to mycobacterial pathogenicity.

## Discussion

Extracellular nucleases from Gram-positive bacterium play vital roles in virulence and degrading DNA in NETs produced by neutrophils^16^ or macrophages^17^. A variety of studies characterizing extracellular nucleases have focused on the proteins that derived from *Streptococcus agalactiae*^6^, *Streptococcus pyogenes*^18^ and *Staphylococcus aureus*^6^,^19^. Though a previous report had described the intracellular nucleases in *M. tuberculosis*^20^, no data is currently available regarding extracellular nucleases from the pathogen. In our investigation, the Rv0888 protein was identified as an extracellular nuclease from *M. tuberculosis* for the first time.

Activity characterization revealed that Rv0888 nuclease was active at temperatures ranging from 37 °C to 51 °C, with an optimal temperature of 41 °C. This temperature range is consistent with the body temperature during fever in humans and animals after *M. tuberculosis* infection, suggesting that Rv0888 may be related to *M. tuberculosis* virulence. Similar to other DNase/nucleases, Rv0888 nuclease requires divalent cations for its activity. Mn^2+^ and Ca^2+^ were found to be optimal for its nuclease activity, although other divalent cations (Mg^2+^, Ba^2+^, Ni^2+^) could also stimulate Rv0888 activity (Table 1). These observations indicated that either the divalent cation diameter was not a factor in accessing the Rv0888 catalytic site or that these cations bind to different amino acids to maintain activity, as previously reported for the *E. coli* nuclease, Colicin E9^21^. Metal chelation, as assessed by EDTA treatment, had a strong inhibitory effect on the activity of the Rv0888 enzyme, suggesting that metal ions were obligatory for nuclease activity and that they might play vital roles in stabilizing the enzymatic structure.

Amino acid substitution studies indicated that the D438 residue may play a key role in Rv0888 nuclease activity, while the H353A and D387A substitutions had a positive influence on Rv0888 activity. The importance of these residues indicate that Rv0888 differs from other bacterial extracellular nucleases which contain the conserved DRGH motif (as in *S. pneumoniae* EndA)^22^ or the degenerate DKGH motif (as in *S. agalactiae* Gbs0661)^6^. Although the Rv0888 protein showed non-specific nuclease activity, it also shares no homology to any known nucleases, as determined by the primary amino acid sequence (Supplementary Fig. S1). Thus, our data suggested that Rv0888 is a novel member of a non-specific nuclease family.

Tuberculosis is difficult to control due to co-infection with HIV and the emergence of virtually untreatable and/or extensively drug-resistant *M. tuberculosis* strains. New drugs and more effective vaccines are urgently needed, thus necessitating a better understanding of the genetic basis for *M.* tuberculosis virulence and pathogenesis^23^. Interestingly, Rv0888 activity was inhibited by four Chinese medicine monomers (Oleuropein, 6-Gingerol, Corylifolinin, and Acteoside). Previous studies have concentrated on small molecule inhibitors, focusing on proteins and factors related to the biosynthesis of pathogens: limiting *S. aureus* entry into endothelial cells by structural analogs of ML 141^24^; blocking *coronavirus* and *filovirus* entry into host cells by the cysteine protease inhibitor K11777^25^; blocking phosphatidylglycerol-LtaS binding and inhibiting LTA synthesis in ItaS-expressin *S. aureus* and in *Escherichia coli* with compound 1771^26^; Inhibiting *A. baumannii* Lipid II (an essential precursor of cell wall biosynthesis) through the use of BAS00127538^27^. Subsequent drug studies then shifted to cell wall proteins and proteases: small molecule inhibitors of sortase that act as an anti-infective therapy against hospital-acquired *S. aureus* infection without the side effects of standard antibiotics^28^; tetrahydrolipstatin that inhibits the phospholipase/thioesterase (Rv3802c) of *M. tuberculosis*^29^; an inhibitor of mPTPB (a virulence factor of tuberculosis) which was acquired by organocatalysis^30^; six small molecules that were confirmed to act as novel EndA (surface endonuclease of the *S. pneumoniae*) inhibitors^31^. Importantly, this manuscript report for the first time that Chinese medicine monomers are effective inhibitors of bacterial nucleases. New drug research is currently mining traditional Chinese medicine to discover novel chemical agents to use against drug-resistant pathogens. Therefore, identification of new virulence factors, and screening their inhibitors from Chinese medicines, may ultimately result in the discovery of novel drugs available to treat TB.

*In vivo* infection experiments suggested that the rMS Rv0888NS/MS and Rv0888S/MS persisted in the lungs of mice infected at 17 d, and histopatholocical analysis revealed that the mouse lungs (7 d post-infection) developed obvious pathological changes, possibly indicating that Rv0888 may act as a virulence factor in *M. tuberculosis* and could relate to the persistence of *M. tuberculosis* in the host. Previous studies have reported different nuclease localizations in bacteria: secreted in the medium (*S. pyogenes*), membrane-associated (*S. pneumoniae*) and peptidoglycan-anchored (*S. pyogenes*, *Streptococcus suis*)^18^,^32^,^33^. In *M. tuberculosis,* Rv0888 contains a signal peptide and is secreted into the medium^14^. This exported product could be more effective for *M. tuberculosis* pathogenecity than a membrane-associated nuclease like *S. pneumoniae* EndA, which would have limited access to its substrate^34^. The characterization of Rv0888 nuclease in this study will facilitate the understanding of the pathogenesis of *M. tuberculosis* and might prompt identification of novel drugs to treat TB.

## Materials and Methods

### Animals, bacterial strains, and growth conditions

The mice used in this study were purchased from Vital River, Beijing, China. All experimental work involving animals was performed according to the guidelines recommended by the animal welfare and ethics of the Heilongjiang Animal Ethics Committee at the Heilongjiang science and technology government agency (Harbin, People’s Republic of China) and was approved and supervised by the commissioner for animal welfare at the Harbin Veterinary Research Institute (HVRI) representing the Institutional Animal Care and Use Committee. All the experiments were designed to minimize the numbers of animals used and every effort was made to minimize both pain and distress to the animals.

*M. smegmatis* cultures were grown in Middlebrook 7H9 medium (BD Biosciences) and supplemented with 10% oleic acid/albumin/catalase enrichment (10% OADC, BD Biosciences), 0.05% Tween 80 (Amresco), and 0.2% glycerol (Sigma-Aldrich). *E. coli* DH5α strain (Novagen) was used for plasmid preparations, and *E. coli* Rosetta 2 strain (Novagen) was used for protein expression. All *E. coli* strains were cultured in Luria-Bertani (LB) medium.

### Cloning and expression

The amino acid sequence of Rv0888 was checked for potential signal sequences using the SignalP 4.0 server (http://www.cbs.dtu.dk/services/SignalP/). Primers (Supplementary Table S1) were designed to amplify the *rv0888*, *rv0888NS*, and *rv0888S* genes from genome of *M. tuberculosis* strain H37Rv. PrimeSTAR Max DNA polymerase (TaKaRa Bio) was used for PCR amplification. The *rv0888* amplicon was cloned into a pET22b vector (Novagen) with a 6 × His-tag at the C-terminal and the recombinant plasmid (pET-22b-Rv0888) was transformed into Rosetta 2 (DE3) *E. coli*. The Rv0888 protein was expressed as the inclusion-body form. The recombinant Rosetta 2 (DE3) *E. coli* was grown in LB medium (200 μg/mL ampicillin and 34 μg/mL chloromycetin) at 37 °C until OD600 = 0.6–1.0, and were then induced with 1 mM IPTG at 37 °C for 4 hours. The *rv0888NS* and *rv0888S* amplicons were cloned into a pMV262 vector (assembled in our laboratory, the corresponding sequence is shown in the supplementary data) and the recombinant plasmids (pMV262-Rv0888NS and pMV262-Rv0888S, respectively) were transformed into *M. smegmatis* mc^2^ 155 cells. The rMS was grown in 7H9 medium (containing 0.05% Tween-80, 0.2% Glycerol, 10% OADC, 50 μg/mL kanamycin) at 37 °C until OD600 = 0.8–1.0. Protein expression was analyzed by SDS-PAGE.

### Site-Directed Mutagenesis

The pET-22b-Rv0888 template plasmid was amplified with complementary mutagenic oligonucleotide pairs (Supplementary Table S2) to introduce N131A, E267A, G303A, H353A, D387A, N389A, D438A, D472A, H473A, D472A-H473A substitutions into the nucleotide sequence; PCR amplification was performed using the PrimeSTAR Max DNA Polymerase (TaKaRa Bio). The PCR products were digested with DpnI to damage the methylated parental template DNA and, the mutated plasmids were transformed into DH5α *E. coli*. All of the substitutions were confirmed by DNA sequencing.

### Purification of Rv0888

Starting from a 4 L bacterial culture, cells were harvested by centrifugation and the pellet was resuspended in 200 mL of buffer A (20 mM Tris-HCl, 150 mM NaCl, 10% glycerol, pH 8.0), and disrupted by a high pressure homogenizer (Constant system USA, 30 kpsi) at 4 °C. Inclusion bodies were isolated from the cell extract by centrifugation at 15,000 rpm for 30 min at 4 °C. The pellet was then washed with 80 mL buffer A, sonicated (350 W, 3 s/3 s, 4 °C) and collected by centrifugation at 15,000 rpm for 30 min at 4 °C. The pellet was suspended with 80 mL 2 M urea by stirring at 4 °C for 4 hours and then centrifuged at 15,000 rpm for 30 min at 4 °C to collect the pellet. Inclusion bodies were solubilized by stirring at 4 °C overnight in 80 mL buffer B (20 mM Tris-HCl, 150 mM NaCl, 6 M urea, 20% glycerol, pH 8.0). The dissolved protein was clarified from insoluble material by centrifugation at 15,000 rpm for 30 min at 4 °C.

The supernatant was dialyzed against 2 L buffer C (20 mM Tris-HCl, 150 mM NaCl, 5 M urea, 20% glycerol, pH 8.0) to 2 L buffer D (20 mM Tris-HCl, 150 mM NaCl, 20% glycerol, pH 8.0) (concentration of urea 5 M–0 M at intervals of 1 M, other conditions remained constant). The dialyzed protein was centrifuged at 15,000 rpm for 30 min at 4 °C. The supernatant was loaded onto a Ni Sepharose 6 Fast Flow resin (GE Healthcare) pre-equilibrated with 10 mL buffer A. Non-binding proteins were washed with 60 mL buffer E (20 mM Tris-HCl, 500 mM NaCl, 10% glycerol, 20 mM imidazole, pH 8.0), 60 mL buffer F (20 mM Tris-HCl, 500 mM NaCl, 10% glycerol, 40 mM imidazole, pH 8.0) and 100 mL buffer G (20 mM Tris-HCl, 1 M NaCl, pH 8.0), and the target protein was eluted with 5 mL buffer H (20 mM Tris-HCl, 150 mM NaCl, 10% glycerol 300 mM imidazole, pH 8.0) and 5 mL buffer I (20 mM Tris-HCl, 150 mM NaCl, 10% glycerol, 500 mM imidazole, pH 8.0), sequentially. The collected proteins were analyzed by SDS-PAGE.

The protein purified from affinity chromatography was centrifuged in a 30 kDa Centricon concentrator (Millipore) and concentrated to ~2 mL. The concentrated protein was loaded onto a HiLoad 16/600 Superdex 200 pg column (GE Healthcare) in buffer J (20 mM Tris-HCl, pH 8.0) and all peak fractions were analyzed by SDS-PAGE. The verified protein was concentrated again and loaded onto a Resource S 5 mL column (GE Healthcare), which was pre-equilibrated with buffer J. The target protein was eluted from the column with a linear gradient of NaCl concentration from 0 M to 2 M. Fractions were collected and analyzed by SDS-PAGE.

### Mass Spectrometry Analysis of Rv0888

The purified protein was run on a 12% SDS-PAGE gel and the lane containing the target protein was removed and cut into pieces of about 1 mm^3^. After washing three times with sterile water, the gel pieces were destained by three sequential 15-min treatments with 100 μL of 25 mM ammonium bicarbonate (Fluka) in 50% (v/v) acetonitrile (Fisher) at pH 8.0, and the pieces where then washed once more with sterile water. The gel slices were dehydrated with 30 μL 100% acetonitrile and completely dried with a Speed-Vac at room temperature. The samples were digested with 8 μL of 0.1 mg/mL trypsin (Promega) overnight at 37 °C. Then, 0.3 μL of the digested protein was mixed with 0.3 μL of matrix solution (5 mg/mL α-cyano-4-hydroxycinnamic-acid (Fluka) in 50% (v/v) acetonitrile (Fisher) and 0.1% (w/v) trifluoroacetic acid (DIMA)) and spotted onto a sample plate for matrix-assisted laser desorption/ionisation time-of-flight mass spectrometry analysis. Positive ion reflection mode was selected and the results were analyzed using 4000 Series software. The target protein was identified by comparing the peptide sequences against homologous proteins in the SwissProt databases using the following parameters: precursor tolerance, ±0.2 Da; MS/MS tolerance, ±0.8 Da; missed cleavages, 1.

### Substrates for Rv0888 nuclease

To measure the substrate specificity for the recombinant Rv0888 nuclease, 0.2 μg of linear dsDNA (PCR amplified), circular plasmid DNA (pGEX-6p-1 vector), chromosomal DNA (*E. coli* DNA) or RNA from baker’s yeast (Sigma-Aldrich) were incubated with 3 μg of the purified Rv0888 protein in 10 μL of reaction buffer (20 mM Tris-HCl, 5 mM MgCl2, pH 7.5) at 37 °C. Buffer (20 mM Tris-HCl pH 7.5) served as a negative control for the Rv0888 protein. After 60 min, 10 μL of the reaction solution was mixed with 1 μL of 10 × loading buffer and analyzed by electrophoresis on a 1.0% agarose gel. All assays were conducted in triplicate.

### Effect of divalent cations and metal chelators on Rv0888 activity

Divalent cations are often required for DNases/nuclease activity^35^,^36^,^37^,^33^. The effects of divalent cations on nuclease activity were determined by incubating the enzyme (20 mM Tris-HCl pH 7.5) with 5 mM of several divalent cation salts (CaCl_2_, MgCl_2_, MnCl_2_, BaCl_2_, NiCl_2_) and various combinations of divalent cation salts (CaCl_2_ + MgCl_2_, CaCl_2_ + MnCl_2_, MgCl_2_ + MnCl_2_) at 37 °C for 1 h. The results were analyzed by electrophoresis on a 1.0% agarose gel. The assays were conducted in triplicate.

### Effect of temperature and pH on Rv0888 activity

The effect of pH on Rv0888 activity was determined using optimal divalent cations over a pH range of 6.0–8.0 (at 0.5 intervals) in 20 mM Tris-HCl at 37 °C for 1 h. The impact of temperature on Rv0888 activity was measured using the optimal divalent cations and the optimal pH over a temperature range of 33 °C–51 °C (at 2 °C intervals) for 1 h. All results were verified by electrophoresis on a 1.0% agarose gel. Rv0888 activity was quantified using a previously described spectrophotometric method^38^. For a 200 μL reaction mixture, 20 μL of 10 × reaction buffer (100 mM Tris-HCl, 50 mM CaCl_2_, 50 mM MnCl_2_, pH 7.5), 20 μL of circular plasmid DNA (100 ng/μL pGEX-6p-1), 10 μL of Rv0888 protein (1 mg/mL), and 150 μL of sterilized water were combined. Next, after incubation at 37 °C for 1 hr, the reaction was stopped by adding 8 μL of 0.5 M EDTA. Then the reaction mixtures were diluted to 2 mL using sterilized water and were measured using a Nanophotometer^TM^ Peal Ultramicro UV-Vis spectrophotometer set at 260 nm. One enzymatic activity unit was defined as the amount of enzyme required to increase the absorbance by 0.001 A260 mL^–1^·min^–1^·cm^–1^ at 41 °C.

### Kinetic studies of Rv0888 nuclease activity

Michaelis-Menton kinetic parameters of Rv0888 were measured using spectrophotometric assays with calf thymus DNA and baker’s yeast RNA. To achieve this, 10 μg of purified Rv0888 protein was incubated at 41 °C with different substrates at varying concentrations (DNA: 0.00625 mg/mL, 0.0125 mg/mL, 0.025 mg/mL, 0.05 mg/mL, 0.1 mg/mL, 0.2 mg/mL, 0.4 mg/mL, 0.6 mg/mL, 0.8 mg/mL; RNA: 0.00625 mg/mL, 0.0125 mg/mL, 0.025 mg/mL, 0.05 mg/mL, 0.1 mg/mL) for 1 h. Rv0888 protein was substituted with 20 mM Tris-HCl (pH 6.5) as a negative control. After incubation, the reactions were stopped by adding an equal volume of 5% ice-cold perchloic acid, followed by dilution to 2 mL with sterilized water. The diluted solutions were measured using a Nanophotometer^TM^ Peal Ultramicro UV-Vis spectrophotometer set at 260 nm. All assays were repeated three times.

### Rv0888-inhibitor screening

Inhibition screening of purified Rv0888 was performed using the following Chinese medicine monomers: Salvianic acid A sodium, Salidroside, dissolved/sterilized Gastrodin and Leonurine; Rhoifolin, Corylifolinin, Cochinchinenin C, Yangonin, Gliquidone, Pterostilbene, Notopterol, Cichoric acid, Loureirin B, Rosiglitazone and Levoalkannim dissolved in 50% dimethyl sulfoxide; Mangostin, Harpagoside and Coniferyl ferulate dissolved in 50% methyl alcohol; Acteoside, Reynoutria japonica Houtt, Echinacoside, Diosmetin, Rutin, Phlorizin, Baicalin, Naringin, Myricetin, Silymarine, Osthole, Gambogic, Neogambogic acid, Rhein, Puerarin, Oleuropein, Chlorogenic acid, Eleutheroside B, 6-Gingerol, Crocin, Curcumin and Resveratrol dissolved in 50% ethanol. All Chinese medicine monomers were purchased from Sigma-Aldrich. 3 μg of purified Rv0888 was pre-incubated at 37 °C with each 0.3 mM of the different Chinese medicine monomers in a final volume of 10 μL. The controls were prepared by substituting the Chinese medicine monomers with sterilized water, 50% dimethyl sulfoxide, 50% methyl alcohol, or 50% ethanol. The remaining activity was determined as described above.

### Lung infection

In this study, 5–6 week old female BALB/c mice (Vital River, Beijing) were maintained under specific pathogen-free conditions and used for lung infection assays. The rMS strains (pMV262, Rv0888NS-pMV262 without its signal peptide, Rv0888S-pMV262 containing its signal peptide) were prepared in 7H9 medium, harvested at full log phase, and washed twice in 2 mL PBS before animal injection. Mice were randomly divided into 4 groups and anaesthetized with diethyl ether prior for bacterial administration. A total of 2 × 10^7 ^cfu of rMS strains (pMV262, Rv0888NS-pMV262, Rv0888S-pMV262) in 50 μL of PBS solution were nasally administered, using PBS as a negative control. At 4 h, 24 h, 4 d, 7 d, and 17 d post infection, 3 mice from each group were killed. Lungs were removed from the mice, with the entire left lobe of the lung homogenized with LB under aseptic conditions and then spread onto LB Agar plates containing 50 μg/mL kanamycin. The plates were incubated at 37 °C for 3–4 days for counting bacteria. About one third of the right lobe of the lung was fixated with 10% formalin for pathological analysis.

Each experiment was independently repeated three times. A two-tailed Student’s t test was used to determine statistical significance, and Prism software (version 5.0; GraphPad, San Diego, CA, USA) was used for these analyses. A *P < *0.05 was considered significant (***P < 0.001).

## Additional Information

**How to cite this article**: Dang, G. *et al.* Characterization of Rv0888, a Novel Extracellular Nuclease from *Mycobacterium tuberculosis*. *Sci. Rep.* **6**, 19033; doi: 10.1038/srep19033 (2016).

## Supplementary Material

Supplementary Information

## Figures and Tables

**Table 1:** Effect of divalent cations on Rv0888 activity.

Divalent cation (5 mM)	Relative activity (%)
None	ND
CaCl_2_	59.89 ± 2.18
MgCl_2_	42.22 ± 2.06
MnCl_2_	78.72 ± 1.84
BaCl_2_	22.10 ± 1.89
NiCl_2_	20.29 ± 1.89
CaCl_2_ + MgCl_2_	61.21 ± 1.95
CaCl_2_ + MnCl_2_	100.00 ± 3.32
MgCl_2_ + MnCl_2_	83.04 ± 3.00

The errors are given as standard deviation. ND: Not detectable.
