# Supplementary material for: Characterization of Rv0888, a Novel Extracellular Nuclease from Mycobacterium tuberculosis
Source: Sci Rep. 2016 Jan 8;6:19033. doi: 10.1038/srep19033 (PMC4733049; doi:10.1038/srep19033)
Supplement: Supplementary Information [file srep19033-s1.doc]

**Characterization of Rv0888, a Novel Extracellular Nuclease from *Mycobacterium tuberculosis***

**Supplementary Information**

Guanghui Dang^a^, Jun Cao^a^, Yingying Cui^a^, Ningning Song^a^, Liping Chen^a^* , Hai Pang^b^*, Siguo Liu^a^*

*^a^Division of Bacterial Diseases, State Key Laboratory of Veterinary Biotechnology, Harbin Veterinary Research Institute, Chinese Academy of Agricultural Sciences, 427 Maduan Street, Nangang Dist, Harbin 15000, PR China.*

*^b^School of Medicine, Tsinghua University, Beijing, 100084, China.*

*Corresponding Author.

E-mail addresses: [lipingchen98@163.com](mailto:lipingchen98@163.com) (L. Chen), pangh@xtal.tsinghua.edu.cn (H. Pang), siguo_liu@hvri.ac.cn (S. Liu).

**Supplementary Tables**

Table S1. Primers used for PCR of Rv0888 genes from *M. tuberculosis*.

| Primer | sequence 5’ 3’ |
| --- | --- |
| Rv0888-F: | CGC**GGATCC**GAGCTCCTCGAGCACC |
| Rv0888-R: | CCG**GAATTC**CGTACGGCCACGTTGTCCGC |
| Rv0888S-F: | TAT**GAATTC**GGATTACGCCAAACGCATC |
| Rv0888S-R: | TAT**CATATG**CCGTACGGCCACGTTGT |
| Rv0888NS-F: | AAA**GAATTC**GCCGACGGATCCGAG |
| Rv0888NS-R: | TAT**CATATG**CCGTACGGCCACGTTGT |
| The restriction sites are indicated in bold. | |

Table S2. The complementary mutagenic oligonucleotides used in this study.

| Primer | sequence 5’ 3’ |
| --- | --- |
| 131N-F | CGACTTCAGCATGCTCACCTACGCCATCGCG |
| 131N-R | GCGGGAAGGGCAGCCCCGCGATGGCGTAGGT |
| 267E-F | GCCTACTACGTCGCGAACGTCCAGGCGGAT |
| 267E-R | TGGTGGTAGGCGAAATCCGCCTGGACGTTC |
| 303G-F | GTGTGCCCTTCTCCGACGCGCTCAATACCC |
| 303G-R | CCTTGAACTCCGAGAGGGTATTGAGCGCGT |
| 353H-F | TGACACGGTCGACGTCTACAACTTAGCTAC |
| 353H-R | CCTCCACCGGTGTTGGTAGCTAAGTTGTAG |
| 387D-F | GTCATCGTCACCGGCGCCTTCAACGCGCGG |
| 387D-R | TGGTCGTCGGAGTACCGCGCGTTGAAGGCG |
| 389N-F | CGGTCATCGTCACCGGCGACTTCGCCGCGC |
| 389N-R | GGTCGTCGGAGTACCGCGCGGCGAAGTCGC |
| 438D-F | CGGCAACGAGTGCGAGCTGCTCGCCAAGAT |
| 438D-R | CCGCTTCGATAGAAGATCTTGGCGAGCAGC |
| 472D-F | TCAATTCCAAGGGTGAGCCACTGTCGGCTC |
| 472D-R | CCACCGCCGGGCTGTGAGCCGACAGTGGCT |
| 473H-F | AAGGGTGAGCCACTGTCGGATGCCAGCCCG |
| 473H-R | GAAGCCGACCACCGCCGGGCTGGCATCCGA |
| 472D-473H-F | CAATTCCAAGGGTGAGCCACTGTCGGCTGCCAGCCC |
| 472D-473H-R | GAAGCCGACCACCGCCGGGCTGGCAGCCGACAGT |

**Supplementary Figure**

**Figure S1. Sequence alignment of Rv0888 with other bacterial extracellular nucleases.** Rv0888, *S. pyogenes* Sda 1 (AAS09918), *L. lactis* YbfB (YP_001031529), *S. pneumoniae* EndA (CAA38134), *S. agalactiae* NucA (NP_735111) peptide sequences were compared by MultAlin software^1^.

Figure S1


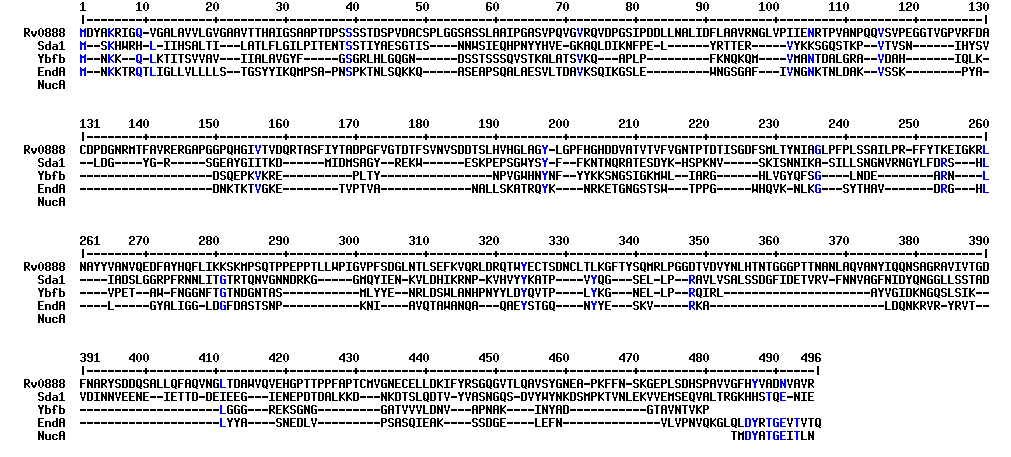


**Supplementary data**

pMV262 vector sequence and multiple cloning site (MCS)

AAAGCCACGTTGTGTCTCAAAATCTCTGATGTTACATTGCACAAGATAAAAATATATCATCATGAACAATAAAACTGTCTGCTTACATAAACAGTAATACAAGGGGTGTTATGAGCCATATTCAACGGGAAACGTCTTGCTCGAGGCCGCGATTAAATTCCAACATGGATGCTGATTTATATGGGTATAAATGGGCTCGCGATAATGTCGGGCAATCAGGTGCGACAATCTATCGCTTGTATGGGAAGCCCCATGCGCCAGAGTTGTTTCTGAAACATGGCAAAGGTAGCGTTGCCAATGATGTTACAGATGAGATGGTCAGACTAAACTGGCTGACGGAATTTATGCCTCTTCCGACCATCAAGCATTTTATCCGTACTCCTGATGATGCATGGTTACTCACCACTGCGATCCCCGGGAAAACAGCATTCCAGGTATTAGAAGAATATCCTGATTCAGGTGAAAATATTGTTGATGCGCTGGCAGTGTTCCTGCGCCGGTTGCATTCGATTCCTGTTTGTAATTGTCCTTTTAACAGCGATCGCGTATTTCGTCTCGCTCAGGCGCAATCACGAATGAATAACGGTTTGGTTGATGCGAGTGATTTTGATGACGAGCGTAATGGCTGGCCTGTTGAACAAGTCTGGAAAGAAATGCATAATCTTTTGCCATTCTCACCGGATTCAGTCGTCACTCATGGTGATTTCTCACTTGATAACCTTATTTTTGACGAGGGGAAATTAATAGGTTGTATTGATGTTGGACGAGTCGGAATCGCAGACCGATACCAGGATCTTGCCATCCTATGGAACTGCCTCGGTGAGTTTTCTCCTTCATTACAGAAACGGCTTTTTCAAAAATATGGTATTGATAATCCTGATATGAATAAATTGCAGTTTCATTTGATGCTCGATGAGTTTTTCTAATCAGAATTGGTTAATTGGTTGTAACACTGGCAGAGCATTACGCTGACTTGACGGGACGGCGGCTTTGTTGAATAAATCGAACTTTTGCTGAGTTGAAGGATCAGATCACGCATCTTCCCGACAACGCAGACCGTTCCGTGGCAAAGCAAAAGTTCAAAATCACCAACTGGTCCACCTACAACAAAGCTCTCATCAACCGTGGCTCCCTCACTTTCTGGCTGGATGATGGGGCGATTCAGGCCTGGTATGAGTCAGCAACACCTTCTTCACGAGGCAGACCTCACTAGTTCCACTGAGCGTCAGACCCCGTAGAAAAGATCAAAGGATCTTCTTGAGATCCTTTTTTTCTGCGCGTAATCTGCTGCTTGCAAACAAAAAAACCACCGCTACCAGCGGTGGTTTGTTTGCCGGATCAAGAGCTACCAACTCTTTTTCCGAAGGTAACTGGCTTCAGCAGAGCGCAGATACCAAATACTGTCCTTCTAGTGTAGCCGTAGTTAGGCCACCACTTCAAGAACTCTGTAGCACCGCCTACATACCTCGCTCTGCTAATCCTGTTACCAGTGGCTGCTGCCAGTGGCGATAAGTCGTGTCTTACCGGGTTGGACTCAAGACGATAGTTACCGGATAAGGCGCAGCGGTCGGGCTGAACGGGGGGTTCGTGCACACAGCCCAGCTTGGAGCGAACGACCTACACCGAACTGAGATACCTACAGCGTGAGCATTGAGAAAGCGCCACGCTTCCCGAAGGGAGAAAGGCGGACAGGTATCCGGTAAGCGGCAGGGTCGGAACAGGAGAGCGCACGAGGGAGCTTCCAGGGGGAAACGCCTGGTATCTTTATAGTCCTGTCGGGTTTCGCCACCTCTGACTTGAGCGTCGATTTTTGTGATGCTCGTCAGGGGGGCGGAGCCTATGGAAAAACGCCAGCAACGCGGCCTTTTTACGGTTCCTGGCCTTTTGCTGGCCTTTTGCTCACATGTTCTTTCCTGCGTTATCCCCTGATTCTGTGGATAACCGTATTACCGCCTTTGAGTGAGCTGATACCGCTCGCCGCAGCCGAACGACCGAGCGCAACGCGTGAGCCCACCAGCTCCGTAAGTTCGGGTGCTGTGTGGCTCGTACCCGCGCATTCAGGCGGCAGGGGGTCTAACGGGTCTAAGGCGGCGTGTACGGCCGCCACAGCGGCTCTTAGCGGCCCGGAAACGTCCTCGAAACGACGCATGTGTTCCTCCTGGTTGGTACAGGTGGTTGGGGGTGCTCGGCTGTCGCTGGTGTTTCATCATCAGGGCTCGACGGGAGAGCGGGGGAGTGTGCAGTTGTGGGGTGGCCCCTCAGCGAAATATCTGACTTGGAGCTCGTGTCGGACCATACACCGGTGATTAATCGTGGTTTATTATCAAGCGTGAGCCACGTCGCCGACGAATTTGAGCAGCTCTGGCTGCCGTACTGGTCCCTGGCAAGCGACGATCTGCTCGAGGGGATCTACCGCCAAAGCCGCGCGTCGGCCCTAGGCCGCCGGTACATCGAGGCGAACCCAACAGCGCTGGCAAACCTGCTGGTCGTGGACGTAGACCATCCAGACGCAGCGCTCCGAGCGCTCAGCGCCCGGGGGTCCCATCCGCTGCCCAACGCGATCGTGGGCAATCGCGCCAACGGCCACGCACACGCAGTGTGGGCACTCAACGCCCCTGTTCCACGCACCGAATACGCGCGGCGTAAGCCGCTCGCATACATGGCGGCGTGCGCCGAAGGCCTTCGGCGCGCCGTCGATGGCGACCGCAGTTACTCAGGCCTCATGACCAAAAACCCCGGCCACATCGCCTGGGAAACGGAATGGCTCCACTCAGATCTCTACACACTCAGCCACATCGAGGCCGAGCTCGGCGCGAACATGCCACCGCCGCGCTGGCGTCAGCAGACCACGTACAAAGCGGCTCCGACGCCGCTAGGGCGGAATTGCGCACTGTTCGATTCCGTCAGGTTGTGGGCCTATCTTCCCGCCCTCATGCGGATCTACCTGCCGACCCGGAACGTGGACGGACTCGGCCGCGCGATCTATGCCGAGTGCCACGCGCGAAACGCCGAATTTCCGTGCAACGACGTGTGTCCCGGACCGCTACCGGACAGCGAGGTCCGCGCCATCGCCAACAGCATTTGGCGTTGGATCACAACCAAGTCGCGCATTTGGGCGGACGGGATCGTGGTCTACGAGGCCACACTCAGTGCGCGCCATGCGGCCATCTCGCGGAAGGGCGCAGCAGCGCGCACGGCGGCGAGCACAGTTGCGCGGCGCGCAAAGTCCGCGTCAGCCATGGAGGCATTGCTATGAGCGACGGCTACAGCGACGGCTACAGCGACGGCTACAACTGGCAGCCGACTGTCCGCAAAAAGCGGCGCGTGACCGCCGCCGAAGGCGCTCGAATCACCGGACTATCCGAACGCCACGTCGTCCGGCTCGTGGCGCAGGAACGCAGCGAGTGGTTCGCCGAGCAGGCTGCACGCCGCGAACGCATCCGCGCCTATCACGACGACGAGGGCCACTCTTGGCCGCAAACGGCCAAACATTTCGGGCTGCATCTGGACACCGTTAAGCGACTCGGCTATCGGGCGAGGAAAGAGCGTGCGGCAGAACAGGAAGCGGCTCAAAAGGCCCACAACGAAGCCGACAATCCACCGCTGTTCTAACGCAATTGGGGAGCGGGTGTCGCGGGGGTTCCGTGGGGGGTTCCGTTGCAACGGGTCGGACAGGTAAAAGTCCTGGTAGACGCTAGTTTTCTGGTTTGGGCCATGCCTGTCTCGTTGCGTGTTTCGTTGCGTCCGTTTTGAATACCAGCCAGACGAGACGGGGTTCTACGAATCTTGGTCGATACCAAGCCATTTCCGCTGAATATCGTGGAGCTCACCGCCAGAATCGGTGGTTGTGGTGATGTACGTGGCGAACTCCGTTGTAGTGCTTGTGGTGGCATCCGTGGCGCGGCCGCGGTACCAGATCTTTAAATCTAGAGGGTGACCACAACGCGCCCGCTTTGATCGGGGACGTCTGCGGCCGACCATTTACGGGTCTTGTTGTCGTTGGCGGTCATGGGCCGAACATACTCACCCGGATCGGAGGGCCGAGGACAAGGTCGAACGAGGGGCATGACCCGGTGCGGGGCTTCTTGCACTCGGCATAGGCGAGTGCTAAGAATAACGTTGGCACTCGCGACCGGTGAGTGCTAGGTCGGGACGGTGAGGCCAGGCCCGTCGTCGCAGCGAGTGGCAGCGAGGACAACTTGAGCCGTCCGTCGCGGGCACTGCGCCCGGCCAGCGTAAGTAGCGGGGTTGCCGTCACCCGGTGACCCCCGTTTCATCCCCGATCCGGAGGAATCACTTCGCA**ATG**GCCAAGACAATTGCGGATCCGTGGTCGCACCCGCAGTTCGAGAAGGGCGGCGGCTCGCAGCTGCA**GAATTC**GAAGCTT**CATATG**ATCGATGTCGACGTGGGCTCGGGC**CACCACCACCACCACCACCACCACCACCAC**GGT**TAA**CTAGCGTACGATCGACTGCCAGGCATCAAATAAAACGAAAGGCTCAGTCGAAAGACTGGGCCTTTCGTTTTATGCCATCATGGCCGCGGTGATCAGCTAGCCACCTGACGTCGGGGGGGGGGG


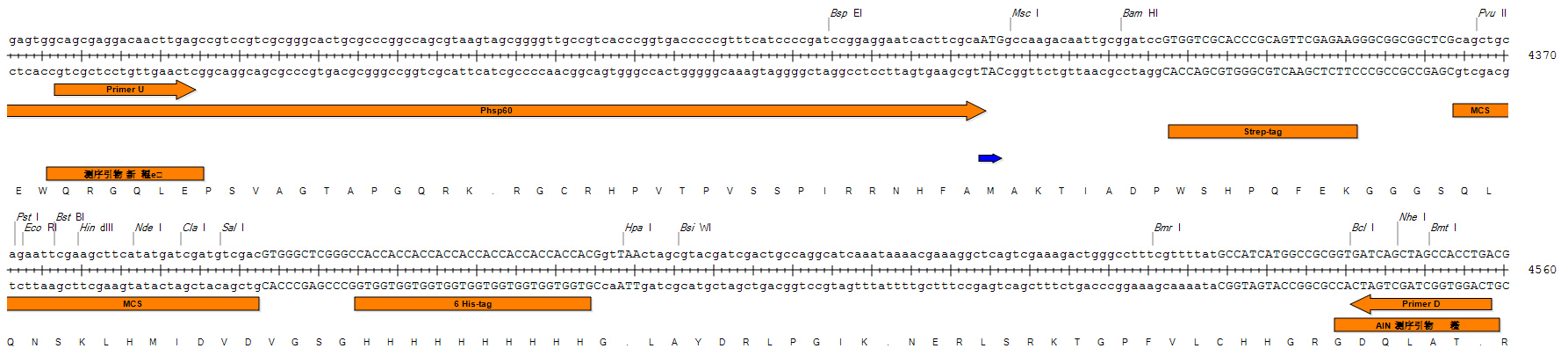


References

1. Corpet, F. Multiple sequence alignment with hierarchical clustering. *Nucleic Acids Res* 16, 10881-10890 (1988).
